# Sexual function of patients with heart failure: distinct phenotypes, distinct sexual function?

**DOI:** 10.1002/ehf2.12182

**Published:** 2017-06-28

**Authors:** Konstantinos Koutsampasopoulos, Antonios Ziakas, Ioannis Vogiatzis

**Affiliations:** ^1^ Department of Cardiology General Hospital of Veria Veria Greece; ^2^ Aristotle University of Thessaloniki 1st Cardiology Dept, AHEPA UGH Thessaloniki Greece

We read with great interest an editorial article on sexual function of patients with heart failure (HF) by Tiny Jaarsma published recently in your journal,^1^ which provided extremely important data regarding the prevalence of sexual problems, the factors related to these problems, and the counselling and treatment for sexual problems in HF patients.

According to the last European Society of Cardiology Guidelines, HF is a clinical syndrome that refers to a wide range of patients: those with HF and preserved ejection fraction (EF) (≥50%—HFpEF), those with an EF in the range of 40–49% (mid‐range HF—HFmrEF), and those with HF and reduced EF (<40%—HFrEF).^2^ Most studies describe high prevalence of sexual problems in patients with HF, but they include patients with HFrEF in which EF and sexual function were not significantly related.^3^ Based on these results, the American Heart Association (AHA) scientific statement on sexual activity and cardiovascular disease advise or discourage sexual activity in accordance with New York Heart Association (NYHA) class regardless of EF.^4^

On the other hand, sexual function in HFpEF and HFmrEF patients has not been studied sufficiently, and existing results of studies comparing sexual function of patients with preserved or decreased left ventricular EF are not representative as they were not designed for HF populations.^5^ For these reasons, it would be very interesting and clinically meaningful to know if there exists any difference in the sexual function between these distinct phenotypes within the heart failure spectrum. This could provide better understanding of the relation between EF and sexual function, alternative explanations of the underlying mechanisms, and possible differentiations in counselling and treatment approach.
